# Telemedicine and delivery of ophthalmic care in rural and remote communities: Drawing from Australian experience

**DOI:** 10.1111/ceo.14147

**Published:** 2022-08-30

**Authors:** Katerina V. Kiburg, Angus Turner, Mingguang He

**Affiliations:** ^1^ Centre for Eye Research Australia Royal Victorian Eye and Ear Hospital East Melbourne Victoria Australia; ^2^ Ophthalmology, Department of Surgery University of Melbourne Melbourne Victoria Australia; ^3^ Lions Outback Vision Lions Eye Institute Nedlands Western Australia Australia; ^4^ Centre for Ophthalmology and Visual Science University of Western Australia Nedlands Western Australia Australia

**Keywords:** Australia, ocular disease, telemedicine

## Abstract

Rural and remote communities in Australia are characterised by small but widely dispersed populations. This has been proven to be a major hurdle in access to medical care services with screening and treatment goals repeatedly being missed. Telemedicine in ophthalmology provides the opportunity to increase the availability of high quality and timely access to healthcare within. Recent years has also seen the introduction of artificial intelligence (AI) in ophthalmology, particularly in the screening of diseases. AI will hopefully increase the number of appropriate referrals, reduce travel time for patients and ensure timely triage given the low number of qualified optometrists and ophthalmologists. Telemedicine and AI has been introduced in a number of countries and has led to tremendous benefits and advantages when compared to standard practices. This paper summarises current practices in telemedicine and AI and the future of this technology in improving patient care in the field of ophthalmology.

## 1 OVERVIEW OF TELEMEDICINE AND ARTIFICIAL INTELLIGENCE EYE‐CARE IN AUSTRALIA

Australia is a large country with a relatively low population density of 3 persons/km^2^, when compared to countries such as the USA (35 persons/km^2^), the UK (265 persons/km^2^) and India (421 persons/km^2^).^1^ To add to challenges around 7 million people (28%) of Australia's population live in rural or remote areas.^2^ It is well documented that people living outside of major cities in Australia are more likely to be diagnosed with a chronic medical condition such as asthma or diabetes, with a greater burden of disease being attributed to individuals living in remote regions. They are also more likely to die at a younger age than individuals in major cities.^2^ Compounding these difficulties, people in remote and very remote regions are more likely to have poor access to health services. With Medicare claims showing that the number of non‐hospital referred attendances (e.g., General Practitioner visits) were lower per person, with 4.8 and 3.6 attendances per person for remote and very remote areas, respectively.^2^ In comparison people living in major cities and inner regional areas had 6.4 visits per person.^3^ Alongside this is a chronic health workforce shortage despite the increased need for medical services. With almost every health profession seeing a decline per 100 000 person with increasing rurality, with the exception of nurses and midwives.^2^ One way of addressing both the higher disease burden and geographic challenge is through the use of telemedicine and more recently the integration of artificial intelligence (AI).

The World Health Organisation defines telemedicine as ‘The delivery of health care services, where distance is a critical factor, by all health care professionals using information and communication technologies for the exchange of valid information for diagnosis, treatment and prevention of disease and injuries, research and evaluation, and for the continuing education of health care providers, all in the interests of advancing the health of individuals and their communities’.^4^ The use of both telemedicine and AI is backed by leading professional bodies with both RANZCO (the Royal Australian and New Zealand College of Ophthalmologists) and Optometry Australia having identified that ‘AI can improve accuracy of diagnosis, lead to better patient outcomes and is likely to play a major role in eye care in the future’. The RANZCO Position Statement in Teleophthalmology in New Zealand from 2020 supported the use of telemedicine; ‘(Teleophthalmology) is a viable means of ensuring the continuity of patient care primary during and immediately after the COVID‐19 pandemic and the rapid expansion of technology in reach, scope and utilisation to meet the needs of society’.^5^ However, they caution that any service should focus on continual improvement to ensure equity, accessibility, effectiveness and efficiency. Telemedicine is well established in ophthalmology, however the introduction of AI in the last 5 years is an emerging component. Current use of AI in Australia is limited to clinical trials and those with a special interest rather than routine clinical care.^6^

Telemedicine has been implemented for comprehensive ophthalmology services. However, one of the most targeted diseases is diabetic retinopathy. Despite improvements in diabetic care, diabetic retinopathy remains the leading cause of visual impairment in working‐age adults.^7^, ^8^ Patients are disproportionately affected in rural and remote communities and individuals that identify as Aboriginal and Torres Strait Islanders.

## 2 OVERVIEW OF AUSTRALIAN TELEOPHTHALMOLOGY IN RURAL AND REMOTE COMMUNITIES

A number of formal telemedicine programs in ophthalmology exist in rural and remote communities across Australia. A recent systematic review of current telemedicine programs in ophthalmology identified five general eye care programs, three emergency care programs and five diseases specific models of care.^9^ There have been two studies examining the use of telemedicine in prisoners requiring ophthalmic assessment.^10^, ^11^ These programs were able to reduce the amount of travel and transfers required for prisoners, with potential economic and security risk benefits. However, since their establishment in the early 2000's have ceased functioning.

An program run in Mt Isa, Queensland, allowed for ophthalmologists working in Townsville to remotely assess patients attending the emergency department, who would have otherwise been required to travel significant distances.^12^ Both patients and doctors were positive about the telemedicine program and a reduced number of acute transfers to Townsville were required, however like the telemedicine for prisoners program this service is no longer functioning.^13^

Lions Outback Vision has provided telemedicine service to rural and remote regions of Western Australia for over a decade. These regions continue to have workforce shortages and telemedicine has enabled increased access for populations dispersed across a vast geographic area.^14^ This service primarily utilises synchronous video consultations and has been highly effective in providing timely access to ophthalmic care by reducing outpatient clinic visits and increasing available specialist time in regions for surgical interventions.^15^ An audit identified the most commonly eye conditions managed which included; red eye, acute vision loss and abnormal retinal photographs.^16^ A number of targeted interventions increased the utilisation of the service 3.5‐fold. These included logistical support, remuneration for optometrists facilitating assessments, improvement of referral pathways and promotion of the telemedicine program.^14^ A qualitative study demonstrated that the majority of patients were either very satisfied or satisfied, with no reports of dissatisfied or very dissatisfied.^17^ Implementation of an on‐call service has increased the participation of Aboriginal and Torres Strait Islanders 10‐fold, due to the infrequent visiting optometry clinics in remote communities necessitating immediate access to specialist rather than future appointment bookings.^18^ An audit of surgery in the regional hospitals found that those patients participating in telehealth had half the waiting time from time of referral to surgical management.^19^

The uptake of telehealth in remote and regional services remains underwhelming given the potential for improving equity in eye care access. The challenges of coordination between outreach optometry and ophthalmology services as well as availability of specialists for on call integration into daily workflow are logistical hurdles.^20^ Future strategies to integrate telehealth into mainstream services require clinical and management service collaboration to clarify local referral and system pathways.

## 3 CURRENT MODALITIES FOR TELEMEDICINE IN OPHTHALMOLOGY IN AUSTRALIA

There are a number of different forms of telemedicine within ophthalmology currently in use, all of which have different logistical and technological requirements (Table 1). The most simple being the acquisition of ocular images such as fundus photographs or optical coherence tomography and subsequent transfer of images to clinicians for asynchronous reporting. This is the most common format for diabetic retinal screening in Australia. More interactive telemedicine systems may include real‐time communication between patient, clinician and specialist via video with relevant ophthalmic imaging acquired preceding or concurrently to the consultation. This requires coordinating availability of clinicians and patients as well as increased technological demands for the transfer of images and video conferencing systems.

**TABLE 1:** Different models of telemedicine and technological requirements

Technology modality	Definition	Technological requirements	Timing
Store and forward	Transfer of patient data (including images and tests)	No communication b/w patient and reviewing clinician. Requires ocular imaging equipment when patient attends	Asynchronous
Real time	Real time communication between patient and specialist at time of appointment with ocular imaging and tests	Stable video/audio conferencing requirement	Synchronous
Hybrid	Patient attends site for required tests prior to telehealth appointment with specialist at home or future time	Equipment for patient exam at site. Stable video/audio conferencing requirement	Asynchronous and Synchronous

## 4 TELEMEDICINE AND EYE CARE IN ABORIGINAL AND TORRES STRAIT ISLANDER COMMUNITIES

Within Australia and internationally Indigenous people have poorer health and social outcomes compared to their non‐Indigenous counterparts. This includes a shorter life expectancy, increased infant mortality and morbidity and lower educational attainment.^21^ Australia is no exception to this trend, with the life expectancy of Aboriginal and Torres Strait Islanders on average 8 years shorter than non‐Indigenous Australians.^22^ When examining eye health related disparities this gap is increased by level of remoteness. With Aboriginal and Torres Strait Islander people aged 40 and above have almost 3 times the rate of vision loss compared to any other Australians.^23^ Australia is the only high‐income country to provide vision impairment data by Indigeneity, providing valuable information on trends and disparities in different communities.^24^ The three main causes of vision loss among Aboriginal and Torres Strait Islander people over 40 were reported in the 2016 National Eye Health Survey and included refractive error (61%), cataract (20%) and diabetic retinopathy (5.2%).^25^ The highest level of screening for diabetic retinopathy is seen in major cities (47%) and inner regional areas (46%) this decreases with increasing rurality. With very remote screening rates decreasing to around 30%.^25^ Of the Indigenous participants in the eye health survey aged 40 and above with a self‐reported diagnosis 53% reported having a diabetic eye exam in the previous 12 months, compared to 78% in non‐indigenous participants aged 50 and above in the past 24 months.^25^ Once diagnosed with diabetic retinopathy, Indigenous Australians are less likely to receive treatment in very remote areas (2%) compared to major cities and outer regional areas (4.2%).^25^

Although resources have been committed to improving screening and treatment rates for ophthalmic conditions in Indigenous populations, between 2008–2009 and 2019–2020 screening rates for diabetic retinopathy only increased by 4%.^25^ A number of barriers exist in order to improve the disparity in disease burden with commonly cited barriers including: reduced access to screening and treatment, distrust of the medical system and a lack of culturally appropriate services and non‐clinical support services.^24^ Although a number of telemedicine models exist within Australia aiming to improve access to care, a lack of culturally appropriate care has also been identified, allowing for the improvement of future service models through the inclusion of Indigenous people in all stages of program design and implementation. This has been highlighted by the United Nations Permanent Forum on Indigenous Issues, who have called for culturally, linguistically and geographically appropriate programs for Indigenous communities as well as increased participation in the design and implementation of these programs.^26^

## 5 EXPANSION OF TELEMEDICINE DURING COVID‐19 PANDEMIC

The COVID‐19 pandemic saw a marked expansion of existing telehealth services and the creation of new of telemedicine programs to maintain both patient and clinician safety. Of great concern during this expansion period was patient safety both from a COVID‐19 perspective and existing chronic health conditions. To minimise the infectious disease risks for both patients and clinicians in Australia, a temporary expansion of Medicare funding for telemedicine during the COVID‐19 pandemic saw the introduction of Medicare benefits to cover the use of audio only consults and the use of telemedicine in metropolitan regions which had previously been unfunded. The lack of funding is often listed as a key reason for a limited uptake of telemedicine. During the COVID‐19 epidemic, ophthalmology saw one of the largest decreases in case volume, with one US study reporting a drop of 81%,^27^ the greatest of any specialty discipline examined. Within ophthalmology the impact to inpatient and outpatient visits was greatest in cataracts (−97%), followed by glaucoma (−88%).^27^ This decrease in both inpatient and outpatient visits is likely to exacerbate already long waitlists for appointments and surgeries without any further intervention.

Of concern during this period of transition was the effect on patient outcomes and safety. Concerns were raised that deteriorating ocular health would be missed and that both patients and clinicians would fail to engage with a telemedicine service. A number of studies have been conducted in order to address this concern and measure any potential impact that a change in patient care may have had. One large meta‐analysis of just under 1000 primary studies, including one on ophthalmology, found that telemedicine in a number of forms was not inferior to conventional care.^28^ Further ophthalmology specific systematic reviews demonstrated that in most cases telemedicine was superior to standard care.^29^, ^30^

## 6 INTEGRATION OF ARTIFICIAL INTELLIGENCE USING DEEP LEARNING SYSTEMS

**FIGURE 1:**
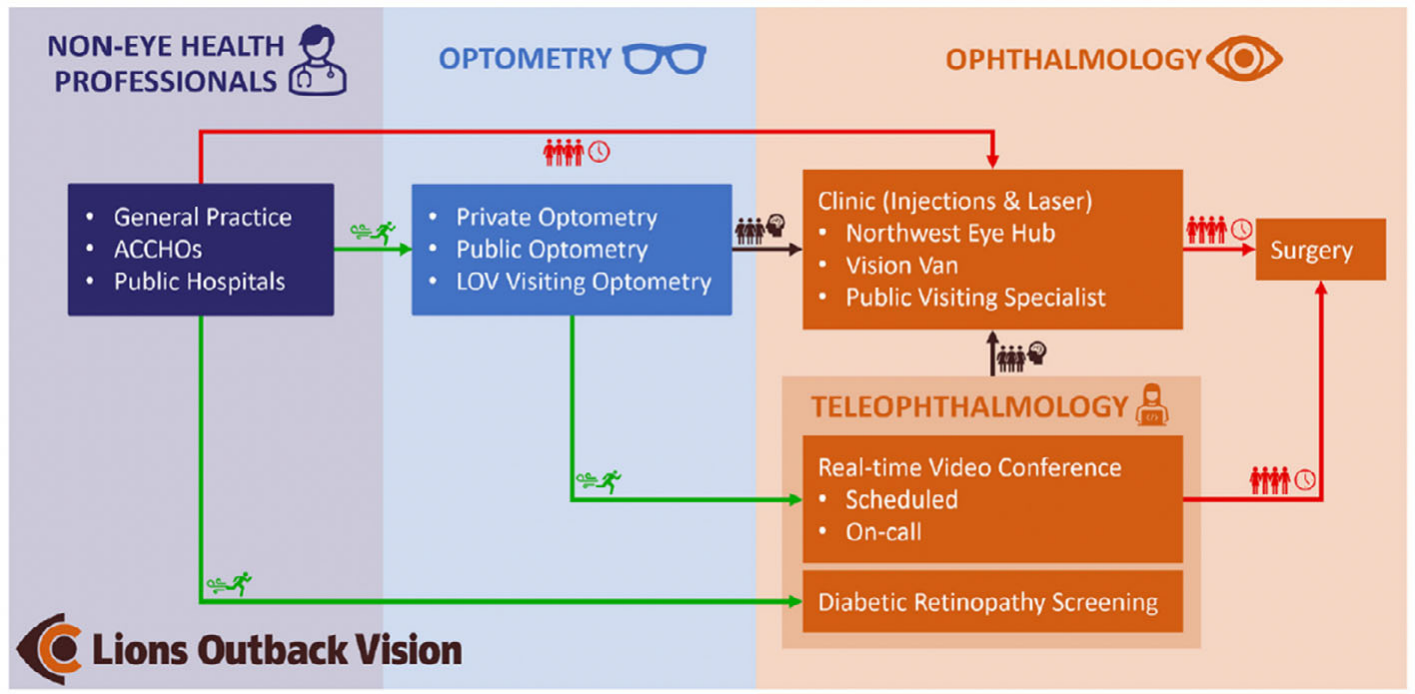
Clinical pathway demonstrating the role of teleophthalmology within Lions Outback Vision. Teleophthalmology enables fast‐track access (green) for patients to specialist care compared to traditional referral pathways which are congested (red). By diverting referrals from non‐eye care professionals via optometry and diabetic retinal screening, specialist clinics can manage well‐triaged pathology (brown). ACCHOs, Aboriginal Community Controlled Health Organisations; LOV, Lions Outback Vision

Clinician triage and assessment is still integral to current models of telemedicine in Australia for rural and remote populations (Figure 1). The increasing burden of eye disease means that communities will require either increased workforce or harness available technology using deep learning systems (DLS). Recent years have seen the introduction of a number DLS for the screening of eye diseases, primary in diabetic retinopathy, with the TGA approving use of two artificial intelligence ocular screening systems in Australia. Globally there are a growing number of diagnostic tools available through other regulatory bodies (Table 2).

**TABLE 2:** Number of DLA solutions currently available

	Approving body	Design	Target disease	Sensitivity/specificity
DR Grader^31^	TGA	CFP	DR	−/92%
EyeArt^32^	FDA, CE	CFP	DR MD GL	92%/92% 90%/70% 90%/93%
Eyetelligence^33^, ^34^, ^35^	TGA	CFP OCT	DR MD GL	92%/94% 100%/93% 96%/92%
IDx‐DR^36^	FDA, CE	CFP	DR	87%/91%
Retinalyze^37^, ^38^	CE	CFP, OCT	DR GL	90%/86% 76%/99%
DeepMind^39^	Research verified	OCT	DR	96%/93%
RetCAD^40^, ^41^	CE	CFP	DR MD	91%/97% 92%/86%
Retmarker^42^	CE	CFP	DR	97%/88%

Abbreviations: CE, Conformitè Europëenne; CFP, Colour Fundus Photography; FDA, Federal Drug Administration; OCT, Optical Coherence Tomography; TGA, Therapeutic Goods Administration.

*Source*: Chia MA, Turner AW. Benefits of Integrating Telemedicine and Artificial Intelligence Into Outreach Eye Care: Stepwise Approach and Future Directions. Frontiers in Medicine.^20^

These DLS are able to triage and assess patients for eye diseases and overcome the initial hurdle of accessing a clinician for assessment which has been identified as a barrier particularly in rural areas. This type of artificial intelligence diabetic retinopathy screening model has been trialled in Indigenous communities in Australia. A trial from our group in a number of specialist and primary care clinics including an Aboriginal Medical Service clinic in Western Australia demonstrated that the sensitivity and specificity were above the above that required by Food and Drug Administration sensitivity and specificity endpoints, 96.9 (95% CI: 89.8, 99.9) and 87.7 (95% CI: 81.8, 92.2), respectively.^43^ More recently our team was awarded a Medical Research Future Fund grant, based upon the previous work using artificial intelligence, in order to develop and clinically validate a fully integrated artificial intelligence system for the early diagnosis of eye and cardiovascular diseases in general practices, endocrinology clinics and cardiology clinics. An integral part of this grant is to examine how to best maximise uptake of the artificial intelligence screening by both patients and clinicians.

Any form of telemedicine is not without its limitations, either in technology and access to clinicians. The introduction of artificial intelligence overcomes the initial hurdle of access to clinicians for screening however these systems are limited by the quality of camera accessible for assessment and the original training image dataset used which tends to be primary of Caucasian descent. This limits the generalisability of artificial intelligence algorithms with area under the curve lower in Aboriginal and Torres Strait Islanders compared to Chinese, Malay and Caucasian populations.^34^ However, these advances in technology provide a promising future for increased access to screening and assessment of eye diseases especially those most disadvantaged communities in rural and remote Australia. It is important that representative populations are included in the development of artificial intelligence algorithms to ensure equity of outcomes for target populations being served.

## 7 ECONOMIC BENEFITS OF INTEGRATING AI

Alongside the benefits of improved and more timely access to care there are economic benefits associated with the use of telemedicine and AI. There is a well‐established evidence base for the cost‐effectiveness of telehealth systems in multiple settings globally.^44^, ^45^, ^46^ Recent studies have examined the introduction of AI as a diagnostic option alongside the more traditional telemedicine format, with even greater potential economic benefits. Economic modelling in Singapore using the national diabetic retinopathy screening program which had 390 006 patients enrolled in 2015, compared the cost of semi‐ and fully‐automatic screening compared to the current human assessment model.^47^ They were able to show the semi‐automated model was the most cost effective ($62 USD per patient per year), followed by the fully automated model ($66 USD per patient per year) with the current human assessment model being the least cost effective ($77 USD per patient per year). Given the expected growth in patients with diabetes requiring diabetic retinopathy screening they estimated savings of 20% of the current annual screening model, which given the estimate of 1 million people with diabetes by 2050 in Singapore would by $15 million.^47^ Although a large proportion of studies to date have examined the use of diabetic retinopathy in telehealth and AI, screening for other diseases such as age‐related macular oedema and glaucoma has also been examined. There appears to be less evidence for the cost benefit in the use of AI in screening for diseases such as age‐related macular oedema and glaucoma with two analyses failing to find a cost benefit. This may be in part due to the relatively large numbers of patients needed to screen to find cases. However, early detection, follow‐up and screening costs also contributed to the high cost.^48^, ^49^ It may be that in the future decreased costs associated with screening and more targeted screening will reverse this cost imbalance, however, the benefit to the individual and community in terms of maintaining sight and ability to continue working and contribute to the economy was not taken into account in these analyses and are a crucial component of disease prevention.

A common theme to these economic analysis is that with greater uptake, the cost‐effectiveness increases. Any new telemedicine program must therefore effectively identify the needs of the population they are serving and integrate into any existing health‐care system in order to maximise the cost benefit both at an individual and government level.

## 8 FUTURE DIRECTIONS

A maldistribution in Australia's eye health clinical workforce means rural and remote populations have chronic issues with eye healthcare access. Therefore, the role of ophthalmologists will be in the management and treatment of surgical management or complex eye diseases. Current telemedicine programs integrate optometrists, nurses and general practitioners; however, further technological advances in ocular imaging and data sharing mean that this can be augmented further and, in some cases, conducted by patients themselves using artificial intelligence.^7^, ^50^

Telemedicine with the addition of artificial intelligence provides many advantages in an aging population with increased healthcare needs, with artificial intelligence screening systems becoming more effective and their integration into telemedicine cheaper. This enhances primary care screening and ensures patients are well triaged for specialist clinician input as required and in an appropriate time frame for optimal visual outcomes.

## 9 CONCLUSION

There are numerous benefits provided by telemedicine ophthalmology programs. These include the increased access to screening and treatment, which is especially important in rural and remote areas which have continue to lag in access and uptake of screening and treatment goals. These telemedicine programs both with and without artificial intelligence enhancements allow for the prompt and early detection of disease, allowing for better patient outcomes. By augmenting screening with integrated artificial intelligence algorithms, further efficiencies may ensure appropriate referrals, reduced travel time and timely triage. A number of studies have already demonstrated the cost effectiveness of telemedicine programs, especially within diabetic retinopathy and these cost savings are likely to increase with the increased expansion and adoption of telemedicine programs. The COVID‐19 pandemic saw the rapid introduction of expanded telemedicine programs in many areas of medicine including ophthalmology. This may serve as an impetus to embrace telemedicine to provide high quality and timely access to healthcare for all Australians no matter their geographic location.
